# Quantification of Parasite Load in Clinical Samples of Leishmaniasis Patients: IL-10 Level Correlates with Parasite Load in Visceral Leishmaniasis

**DOI:** 10.1371/journal.pone.0010107

**Published:** 2010-04-09

**Authors:** Sandeep Verma, Rajesh Kumar, Gajendra Kumar Katara, Laishram Chandreshwor Singh, Narender Singh Negi, V. Ramesh, Poonam Salotra

**Affiliations:** 1 Institute of Pathology (ICMR), Safdarjung Hospital Campus, New Delhi, India; 2 Department of Dermatology, Safdarjung Hospital, New Delhi, India; 3 Department of Medicine, Safdarjung Hospital, New Delhi, India; Federal University of São Paulo, Brazil

## Abstract

A rapid and accurate method to detect and quantify *Leishmania* parasite is urgently needed to facilitate early diagnosis of Leishmaniasis and monitoring of antileishmania therapy. In this study, real-time assay was applied to estimate parasite load in clinical samples of visceral leishmaniasis (VL) and post kala-azar dermal leishmaniasis (PKDL) patients. The mean parasite load in blood of VL patients (n = 31) was 8,372 parasites/ml, while the mean parasite load in bone marrow aspirate (BMA) was 194,962 parasites/million nucleated cells (n = 12). Parasite load was undetectable after treatment with amphotericin B (n = 9) in VL, while a residual parasite burden was detected in 2 of 6 patients following treatment with sodium antimony gluconate. Further, circulating levels of IFN-γ, TNF-α, IL-10, IL-6, IL-4 and IL-2 were analysed in VL patients (n = 29) by Cytometric Bead Array to evaluate correlation with parasitic load. Interestingly, IL-10 levels correlated significantly with parasite load (r = 0.82, *P*<0.0001). The mean parasite load in dermal lesions of PKDL patients was 9,502 parasites/µg tissue DNA at pre-treatment stage (n = 25), with no detectable parasites after therapy (n = 5). Parasite burden was distinctly higher (*P*<0.0001) in nodular lesions (n = 12) (19,586 parasites/µg tissue DNA) compared to papular/macular lesions (n = 13, 193 parasites/µg tissue DNA). Further, chronic PKDL lesions showed significantly (*P* = 0.0166) higher parasite load in comparison with acute lesions. Results indicate that chronic, nodular cases constitute the major parasite reservoir for anthroponotic transmission. Our results establish that the high parasite load in VL is strongly correlated with a high level of IL-10, implicating IL-10 as a marker of disease severity. The assay is applicable for diagnosis as well as prognosis of both VL and PKDL, providing a simple molecular tool to monitor the efficacy of antileishmanial drugs or vaccines.

## Introduction

The protozoan parasites of the genus *Leishmania* are the causative agents of a group of diseases called leishmaniasis, endemic in more than 88 countries and affecting 12 million people worldwide. Depending on the species, *Leishmania* produce a wide spectrum of diseases, from simple cutaneous, mucocutaneous to deadly visceral leishmaniasis (VL). The visceral form of the disease, caused by *L. donovani*, *L. infantum*, and *L. chagasi*, is prevalent in tropical regions, with more than 90% of total cases reported from Bangladesh, Brazil, India, and Sudan [1]. In India, the state of Bihar, parts of Eastern Uttar Pradesh, and West Bengal are endemic foci of VL where periodic epidemics are common claiming the lives of thousands and causing severe morbidity to hundreds of thousands [2]. This burden is further compounded in India as about 5–15% of apparently cured VL patients develop an unusual dermal form of the disease termed Post kala azar dermal leishmaniasis (PKDL) [3]. The need to search for cases of PKDL and treat them as a part of VL control program has been emphasized since patients with PKDL provide the only known reservoir for the parasite during inter-epidemic periods of VL in India [4].

Current diagnostic methods based on parasite detection (stained smears, culture or histopathology) are invasive and have poor sensitivity, while immunological methods (Direct Agglutination Test, enzyme-linked immunosorbent assay etc.) have limited specificity, fail to distinguish between past and present infections because of persistence of antibodies, and are not reliable in immune-compromised patients [5]. In recent years, PCR has been applied successfully to detect *Leishmania* spp. in cases with any of the clinical manifestations of leishmaniasis. Several PCR protocols for combined detection and differentiation of parasites exist, including multiplex PCR [6], PCR plus sequencing [7], and restriction fragment length polymorphism (RFLP) analysis [8]. However, the multiple steps of post-PCR manipulation in these procedures require time and pose the risk of DNA contamination. Rapid and accurate methods for parasite detection and monitoring parasite load in VL and PKDL lesion tissues would greatly enhance the clinical management of the disease. Real-time PCR is reported as a promising tool for detection and quantification of various parasites including *Toxoplasma gondii* [9], *Trypanosoma cruzi* [10] and *Plasmodium* spp. [11]. Previously, quantification of *Leishmania* spp. in mouse tissues was determined using TaqMan PCR method [12], [13] or SYBR Green real-time assay [14]. There are limited reports using real-time PCR for diagnosis and quantification of *Leishmania* spps in humans [15]–[19], while such studies are lacking in human VL and PKDL caused by *L. donovani*.

It is of interest to evaluate the association of parasitic burden with immune response in human VL, since immune modulators are considered to play a major role in the disease pathogenesis [20]. In murine model, it is generally accepted that Th1 type response is needed for control and protection against *Leishmania* infections, and interferon gamma (IFN-γ) secreted by Th1 cells, is the most potent macrophage-activating cytokine leading to host resistance to infection with *Leishmania* parasites [21]. Human studies on immune response in VL have shown increased levels of serum IFN-γ, tumor necrosis factor alpha (TNF-α), interleukin (IL)-1, IL-6, and IL-10 [22]–[26], however, the presence of high levels of both IFN-γ and IL-10 during active VL creates a puzzle in understanding the disease pathogenesis [27], [28].

In the present study, real time PCR assay was established for diagnosis and measuring parasite load in VL and PKDL patients. The study was extended to measure the parasite burden at post treatment stage in order to monitor the efficacy of treatment. We demonstrate a strong correlation of parasite burden with the host immune response in VL and the pattern of parasite load with respect to the clinical presentation in PKDL.

## Materials and Methods

### Patients and samples

Patients of VL and PKDL originated from Bihar and reported to Departments of Medicine or Dermatology, Safdarjung Hospital, New Delhi were included in this study. Confirmed VL cases (either LD positive in BMA or PCR positive) and PKDL patients (LD positive or PCR positive) were included in this study. The profile of patients in the study is described in Table 1 and 2. Blood samples (n = 20) from healthy volunteers and dermal tissues from leprosy patients (n = 15) were collected as controls. Patients of VL were given treatment with SAG (20 mg/kg/day) for 28 days or Amp B (0.75–1.0 mg/kg/day) for 15—20 infusions on alternate days. PKDL cases were treated with SAG (20 mg/kg/day) for 4 months. Clinical samples were taken before starting treatment and one day after the last dose of treatment.

**Table 1:** Results of real-time PCR assay for *Leishmania* DNA in blood samples of VL patients.

Patient no.	Age/Sex	Microscopy with BMA	Region	Ct±S.D.	Parasite load/ml Blood
1	24/M	N	H	29.70±0.24	39
2	42/M	N	L	29.47±0.36	39
3	23/F	N	H	29.31±0.3	43
4	6/M	N	L	29.17±0.49	48
5	18/F	ND	L	28.81±0.28	60
6	42/F	P	H	28.17±0.36	92
7	16/M	N	H	27.65±0.37	131
8	11/M	N	H	27.31±0.67	164
9	7/M	N	L	27.24±0.18	171
10	13/M	P	H	27.23±0.31	172
11	25/F	P	L	27.22±0.23	173
12	12/M	P	H	26.83±0.15	225
13	48/M	P	L	26.55±0.16	270
14	17/F	N	H	26.50±1.09	281
15	63/M	P	H	26.34±0.43	312
16	17/M	N	L	26.23±1.0	336
17	12/M	P	L	26.07±0.66	372
18	4/F	N	H	26.05±0.14	377
19	15/M	N	L	25.97±0.59	399
20	4/M	P	H	25.79±0.77	450
21	11/M	N	L	23.59±0.33	1939
22	8/F	P	H	23.04±0.87	2801
23	6/F	P	L	22.87±0.50	3130
24	7/M	P	H	22.85±0.38	3165
25	12/M	P	H	22.63±0.70	3663
26	35/M	P	H	22.61±0.31	3729
27	7/M	P	L	22.49±0.8	4021
28	40/F	P	H	21.91±0.30	5939
29	19/M	P	L	21.84±0.45	6195
30	9/F	N	H	21.25±0.19	9171
31	14/F	P	H	16.53±0.48	211613

M- Male, F-Female, P- Positive,N- Negative, ND- Not Determined, H- region of High.

endemicity, L- region of Low endemicity, Ct- Threshold cycle, S.D.- Standard deviation.

**Table 2:** Results of real-time PCR assay for *Leishmania* DNA in BMA samples of VL patients.

S. no.	Patient no.	Microscopy with BMA	Region	Parasite load per million nucleated cells
1	2	N	L	431
2	3	N	H	715
3	4	N	L	106
4	7	N	H	332
5	10	P	H	2849
6	13	P	L	53818
7	14	N	H	444518
8	15	P	H	1091315
9	16	N	L	23772
10	17	P	L	31857
11	19	N	L	6720
12	26	P	H	683106

N-Negative, P-Positive, H- region of High endemicity, L- region of Low endemicity.

### Ethics Statement

The study was approved by and carried out under the guidelines of the Ethical Committee of the Safdarjung Hospital, India. All patients or responsible adults provided written informed consent for the collection of samples and subsequent analysis.

### DNA isolation

Blood and bone marrow sample was collected in heparinized tubes. DNA extraction was performed by using a QIAamp DNA Blood mini kit (Qiagen, Germany) according to manufacturer's instructions. In order to achieve maximum yield, digestion was performed overnight with proteinase K in Qiagen lysis solution ALS. DNA was isolated from 200 µL of blood and eluted in 50 µL distilled water. In case of BMA sample, 100 µL of BMA was used and eluted in 100 µL distilled water. PKDL tissue was collected in NET buffer [150 mM NaCl, 15 mM Tris-HCl (pH 8.30) and 1 mM EDTA]. Tissue was homogenized using liquid nitrogen with pestle and mortar. DNA extraction from homogenized tissue was performed by using QIAamp DNA Tissue kit according to manufacturer's instructions. DNA was eluted in 100 µL distilled water. All samples were processed on the same day and DNA was stored at 80°C until use.

### Real-time PCR assay

Real-time PCR based on SYBR Green I was applied for accurate quantification of the target sequence in the current study. Genus specific primers based on kDNA were designed by using Primer Express software 2.0 (Applied Biosystems, USA) and consisted of 5′-CTTTTCTGGTCCTCCGGGTAGG-3′ (forward), and 5′-CCACCCGGCCCTATTTTACACCAA-3′ (reverse). These primers were found to match with those used in the quantification study of *L. infantum* DNA in blood samples of VL by a real-time PCR assay based on TaqMan probe [16]. A standard curve was constructed using 10-fold serially diluted *L. donovani* parasite DNA corresponding to 10^4^ to 0.1 parasite per reaction. Amplification and detection were performed using an ABI Prism 7000 sequence detection system (Applied Biosystems, USA). Standards, samples, and negative controls were analyzed in triplicate for each run. A 10 µl of the PCR reaction was performed, consisting of 1× SYBR Green I PCR Master mix (Applied Biosystems, USA), 5 pmol forward primer, 5 pmol reverse primer, and 1 µl volume of DNA from the blood, BMA and tissue sample. Cycling parameters were 50°C for 2 min, 95°C for 10 min, and 40 cycles at 95°C for 15 s and 60°C for 1 min. A threshold cycle value (Ct) was calculated for each sample by determining the point at which the fluorescence exceeded the threshold limit. A standard curve was obtained by plotting the Ct values against each standard of known concentration parasite DNA. Each real time PCR reaction was carried out in triplicate.

### Quantification of the human albumin gene in BMA samples

In order to allow a comparison of parasite loads between the different BMA samples, we quantified the number of nucleated human cells using housekeeping gene (Albumin). We used primers, forward- 5′ GCT GTC ATC TCT TGT GGG CTG T 3′ (100 pmol) and reverse- 5′GGA GAG ATT TGT GTG GGC ATG ACA 3′ (100 pmol) and thermal profile was identical to those of kinetoplast DNA amplification [16]. The standard curve was established from DNA extracted from the THP-1 human monocytic cell line.

### Cytokine measurement

Whole blood, collected in sterile tubes at pre-treatment stage, was allowed to coagulate for 2 to 3 h at 4°C prior to centrifugation. Sera were preserved at −70°C until cytokine measurement was performed using Cytometeric bead array (CBA) kit (BD Biosciences, USA) as per manufacturer's instructions. Briefly, 50 µl of bead populations with discrete fluorescence intensities of Peridinin chlorophyll protein (PerCP)-Cy5.5 and coated with cytokine specific capture antibodies was added to 50 µl of patient sera, and 50 µl of phycoerythrin-conjugated anti-human Th1/Th2 cytokine antibodies. Simultaneously, standards for each cytokine (0–5000 pg/ml) were likewise mixed with cytokine capture beads and phycoerythrin-conjugated reagent. The vortexed mixtures were allowed to incubate for 3 hrs. Beads were washed and analyzed using flowcytometry (FACS Calibur, BD Biosciences, USA). Quantity (pg/ml) of respective cytokine was calculated using CBA software. Standard curves were derived from the cytokine standards supplied with the kit. The lower limit of detection ranged from 1 to 2.1 pg/ml for different cytokines.

### Statistical analysis

The differences between experimental groups were analyzed using the Mann-Whitney test. Correlation was evaluated using Spearman/Pearson correlation test. All data are presented as mean ± SD and a difference in mean values was considered significant when the *P* value was <0.05.

## Results

### Sensitivity and linearity of the Real-time PCR assay

**Figure 1:**
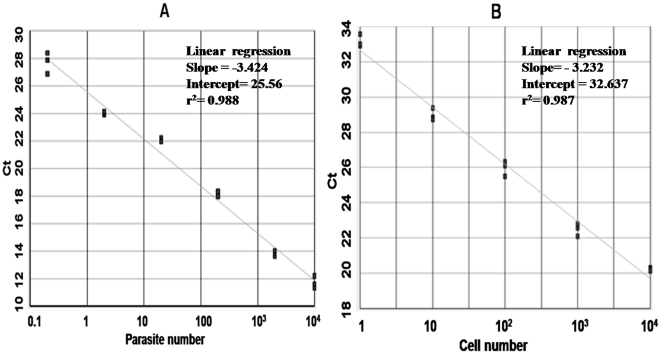
Standard curves obtained with SYBR Green 1 based real time PCR. (A) Amplification of *Leishmania* DNA expressed as the number of parasites per reaction. (B) Amplification of THP-1 cell line DNA expressed as the number of human nucleated cells per reaction.

Initially, the sensitivity of the assay was determined by serially diluted known amount of parasite DNA (10 ng – 1fg). The reaction allowed a detection limit of 1 fg DNA, corresponding to 0.01 parasite (data not shown), permitting the detection of 0.01 parasite per reaction. The mean standard curve was linear over 6 log range of DNA concentrations with correlation coefficient (r^2^) of 0.988 (Fig 1A). The intra-assay coefficient of variations of Ct values among the replicates was 3.92, 1.44, 1.37, 0.67, 0.41 and 0.72 for six different concentrations. The mean standard curve with DNA from THP-1 cells was also linear over 5 log range of DNA concentrations with a correlation coefficient (r^2^) of 0.987 (Fig 1B). A negative control (water instead of template DNA) with each PCR assay was included for stringent measures to control contamination.

### Analysis of parasite load in Blood and BMA of VL patients

Evaluation of parasite load was carried out in clinical samples of VL patients at pre-treatment (n = 31) and post-treatment stage (n = 15). At the time of diagnosis, the mean parasite load in blood was 8,372 parasites/ml with a wide range of 39 parasites/ml to 2.1×10^5^ parasites/ml (Table 1). There was no parasite detected in blood samples from healthy persons (n = 20). Evaluation of parasite load in BMA (n = 12) indicated a mean value of 194,962 parasites/million nucleated cells with a range of 106 parasites to 10.9×10^5^ parasites/million nucleated cells (Table 2). Parasite levels in blood and BMA showed a good correlation (r = 0.76). The microscopically positive cases had significantly higher (*P* = 0.0152) parasite load in blood (mean = 14,490 parasites/ml) in comparison with negative cases (mean = 1,010 parasites/ml). Likewise microscopically positive cases had significantly higher (*P* = 0.0480) parasite load in BMA (mean = 372,589 parasites/million nucleated cells) than microscopically negative cases (mean = 68,085 parasites/million nucleated cells). There was no significant difference in parasite load in either blood (*P* = 0.6309) or BMA (*P* = 0.4848) between cases from low and high endemic regions.

Further, parasite load was also determined after treatment in patients treated with SAG (n = 6) or Amp B (n = 9). At post-treatment stage, parasites were detected in blood samples of 2 patients (no. 23 and 25), both treated with SAG, with parasite load as 166 and 415 parasites/ml respectively. Microscopy with BMA at post-treatment stage was negative for patient 23 but positive for patient 25 who was further treated with Amp B.

### Parasite load in PKDL tissues

**Figure 2:**
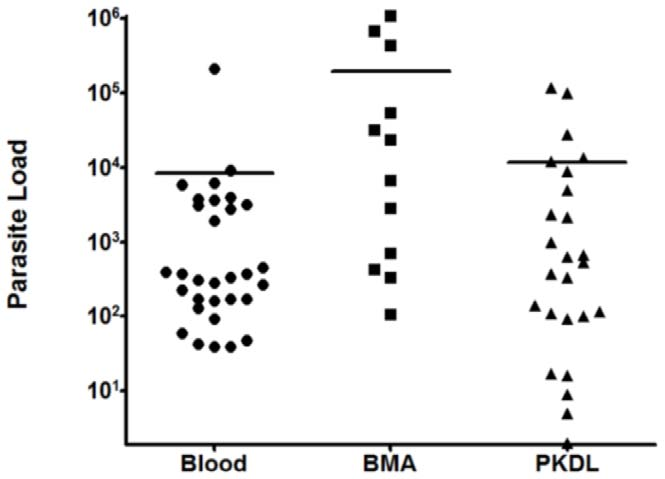
Scatter plot showing parasite load in Blood, BMA and PKDL samples. Parasite load in VL blood samples (Parasites/ml blood) and in BMA samples (Parasites/million nucleated cells) and in PKDL lesion tissues (parasites/µg tissue DNA) were determined by real-time PCR. Horizontal line represents the mean parasite load.

*Leishmania* DNA was detected in lesion tissue of all the 25 patients of PKDL. The mean value of parasite load was 9,502 parasites/µg tissue DNA with range of 2 to 144,586 parasites/µg tissue DNA (Table 3), while all leprosy lesions (n = 15) were negative. Comparative assessment of parasite load in various clinical polymorphic forms of PKDL revealed significantly higher (*P*<0.0001) parasite load in nodular lesions (n = 12) with a mean of 19,586 parasites/µg tissue DNA as compared to parasite load in papular/macular lesions (n = 13), which had a mean of 193 parasites/µg tissue DNA. Further, correlation of parasite load was also evaluated with the chronicity of PKDL lesions. There was significantly (*P* = 0.0166) lower parasite load in acute cases with history of less than 3 years (n = 12, mean = 440 parasites/µg tissue DNA) in comparison with chronic cases with history of more than 3 years (n = 13, mean = 17,867 parasites/µg tissue DNA). Overall, real-time was positive in all the patients (100%), while slit smear microscopy showed 50% positivity. Further, direct microscopy was differentially analyzed in various polymorphic forms of PKDL such as papular/macular and nodular lesions. Positivity for slit smear in papular/macular and nodular was 38% and 63% respectively. The parasite load was comparable in patients from region of low and high endemicity (*P* = 0.5187). There was no parasite detected in tissue samples taken at post treatment stage (n = 5). Scatter plot of parasite load in VL (Blood, BMA) and PKDL lesion tissues is shown in Fig 2.

**Table 3:** Clinical presentation and results of real-time PCR assay in dermal lesions of PKDL patients.

Patient no.	Age/Sex	Region	Clinical presentation	Duration of chronicity	Slit Smear	Ct±S.D.	Parasite Load/µg tissue DNA
1	45/M	H	Papular	2 Months	N	25.02±0.12	39
2	10/M	H	Papular	6 Months	N	22.44±0.07	82
3	25/M	H	Papular	7 Months	N	21.96±0.38	50
4	12/M	H	Macular	1.5 Year	P	22.69±0.64	82
5	20/F	H	Papular	2 Years	P	22.25±0.23	105
6	16/M	L	Macular	3 Years	N	25.61±0.45	14
7	65/M	H	Papular	3 Years	N	25.15±0.96	2
8	18/M	H	Papular	3 Years	P	23.82±1.29	39
9	60/M	H	Papular	3 Years	N	19.55±0.76	603
10	21/M	H	Papular	4 Years	N	25.71±1.24	11
11	19/M	L	Papular	4 Years	P	21.79±0.29	461
12	18/M	H	Papular	6 Years	P	21.64±0.53	1022
13	22/M	H	Papular	10 Years	N	28.73±0.59	2
14	19/M	L	Nodular	1.5 Years	P	19.58±0.04	522
15	30/F	H	Nodular	2 Years	N	16.43±0.23	3022
16	14/M	H	Nodular	2.5 Years	P	19.48±0.98	719
17	17/M	H	Nodular	4 Years	P	20.13±0.07	966
18	27/M	L	Nodular	4 Years	P	13.43±0.20	144586
19	35/M	H	Nodular	6 Years	P	19.15±0.40	499
20	35/M	H	Nodular	6 Years	P	16.30±0.82	5180
21	28/M	H	Nodular	6 Years	N	16.62±0.52	3926
22	28/M	L	Nodular	7 Years	N	16.57±1.26	1512
23	52/M	H	Nodular	7 Years	ND	16.18±0.60	14870
24	19/M	H	Nodular	7 Years	N	15.36±0.37	12945
25	26/F	H	Nodular	10 Years	P	11.61±0.75	46286

M- Male, F-Female, P- Positive, N- Negative, ND- Not Determined, H- region of High endemicity, L- region of low endemicity.

### Correlation of immune response with parasite load

**Figure 3:**
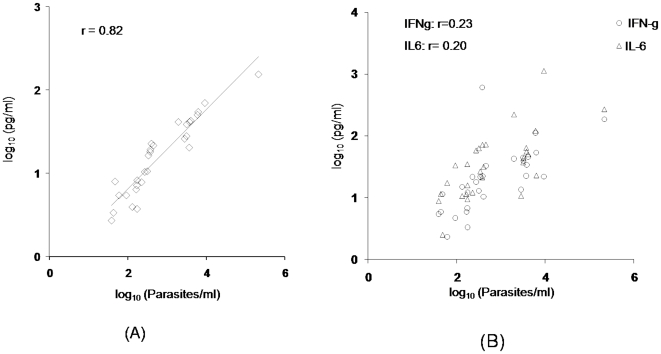
Comparative assessment of IL-10, IFN-γ, IL-6 and parasitic load in blood samples of VL patients. The serum levels (pg/ml) of IL10 (Panel A) and IFN-γ and IL-6 (Panel B) were measured by CBA and parasite load (Parasites/ml) determined by real-time PCR. Diagonal line represents linear regression.

Expression levels of IFN-γ, TNF-α, IL-10, IL-6, IL-4 and IL-2 were analyzed in sera of VL patients (n = 29) by CBA. The level of IL-10 was significantly higher (*P*<0.0001) in cases with high parasite load with correlation coefficient r = 0.82 (Fig. 3A). No correlation with parasite load was observed for other cytokines examined. The levels of two major cytokines implicated in VL, IFN-γ and IL-6, with respect to parasite load are shown in Fig 3B.

## Discussion

Reliable and rapid diagnostic and prognostic tools are desperately needed for an effective control of VL. The sensitivity, specificity and reproducibility of our PCR assay were comparable or better as compared to other real time PCR studies [15]–[19]. Reproducibility of the results was high as assessed through estimation of mean Ct values, SDs and intra-assay coefficient of variations. In the current study, a wide range of parasite burden was observed in peripheral blood as well as BMA of VL patients irrespective of their origin from areas of high or low endemicity. The real-time PCR assay was positive in all VL and PKDL cases while direct microscopy was positive in 57% VL cases and 50% PKDL cases. We observed that VL patients with low parasitic load were often negative in microscopic examination. Being more economical as compare to TaqMan based real-time PCR, the assay is applicable as a rapid, reliable diagnostic and prognostic tool in reference labs for samples from remote or poorly equipped endemic areas.

A wide range of parasite burden was observed in dermal tissues of PKDL patients with nodular lesions showing significantly higher parasite load in comparison with macular/papular lesions. Previously, it has been reported that *Leishmania* parasites are scanty and difficult to demonstrate in skin lesions with macular/papular presentation [29]. The current study confirmed these observations and presented quantification of parasite burden by real-time PCR which is more accurate as compared to microscopic assessment. Direct microscopy with slit smears of PKDL lesions is reported to have low sensitivity (13–60%) [30]. We made similar observations in slit smear of PKDL patients with 38% positivity in patients with macular/papular and 63% in nodular lesions. Under such circumstances, establishing the diagnosis of PKDL and differentiating it from leprosy by traditional methods becomes difficult particularly for macular/papular cases and real time PCR assay provides a reliable alternative, being faster than routine PCR. Further, parasitic load was substantially higher in chronic lesions as compared to acute lesions suggesting that parasitic load is increasing with time. This is in contrast to the situation in CL where parasite load is higher in acute lesions in comparison to late lesions [31]. The present data indicates that chronic/nodular skin lesions rich in LD bodies, may be the main source of vector infection in the community particularly in the absence of animal reservoir, and play an important role in the disease transmission from humans to the insect.

Clinical outcome of the disease is a consequence of the complex interaction between the pathogen and the host and survival of pathogen largely depends on the type of cytokine (Th1 or Th2) being produced by host immune cells on encounter. We therefore analyzed the major Th1/Th2 cytokines in VL patients to understand the correlation of immune response with parasite load. Our study revealed significantly elevated IL-10 levels, strongly correlating with increasing parasitic load in VL patients. An extreme case with unusually high IL-10 level also presented with the highest parasite load. Similar observation was reported earlier with *L. donovani* infection in murine models where it was found that IL-4 and IL-10 might act in concert to ensure logarithmic parasite growth [32]. In canine VL, expression of IFN-γ, TNF-α, IL-10 and TGF-β in lymph nodes associated with parasite load [20]. A recent study in canine VL has shown association between expression level of IFN-γ and IL-4 with parasite load suggesting them as prognostic markers for monitoring disease progression [33]. Further, the same group has shown a correlation of parasite load with clinical manifestation of the disease in canine VL [34]. We found a striking association of IL-10 with parasite load suggesting that IL-10 levels may be correlated with disease severity in human VL. We have recently documented a significant correlation between IL-4 expression and parasite load in cutaneous leishmaniasis patients suggesting high IL-4 expression may help in parasite survival by suppressing host immune responses at the site of infection [31]. Functionally IL-10 is well described as a suppressive or deactivating cytokine; *in vitro*, it has been shown to inhibit antigen presentation [35], antigen-specific T cell proliferation and type 1 cytokine production [36] and to render macrophages refractory to activation by IFN-γ for intracellular killing [37]. The endogenous production of IL-10 has been shown to hinder the clearance of many other infectious organisms including *Klebsiella pneumoniae*, *Brucella abortus*, *Candida albicans*, *Trypanosoma cruzi* and *Mycobacterium avium* [38]–[42]. Role of IL-10 in parasite persistence and establishing latency associated with natural infection has been demonstrated using knockout mice [43]. Previous studies including ours have documented elevated levels of IL-10 in VL patients [23], [28], [44]–[46] and its association with pathology [27], [47]. Thus, IL-10 is well established as a critical component in susceptibility to *Leishmania* infection that contributes to the pathogenesis of VL by inhibiting the cytokine mediated activation of immune responses. Our data demonstrates that IL-10 production, rather than any other cytokine, is a clear correlate of severity of VL in human.

At post-treatment stage, parasites were undetectable in a majority (87%) of VL patients; however, 2 out of 6 patients treated with SAG had residual parasite in blood. One of these, with parasite load as 415 parasites/ml after therapy, was found positive with BMA microscopy and was further treated with Amp B, while the other was lost to follow up. Our data are in agreement with the reports that treatment failure is often associated with SAG treatment, where most alarming reports came from Bihar, India [48] that responded only to Amp B [49]. Therefore, the assay could help in early detection of treatment failures and offers an opportunity for point-of-care prognostic evaluation and monitoring the efficacy of chemotherapy.

This study emphasizes that IL-10 production correlates with parasitic burden in active human VL, making it a biomarker of disease severity. Chronic/nodular cases of PKDL are demonstrated to have a high parasitic load, thus constituting the main reservoir of the parasite in India. The real-time PCR assay has immense application value as a rapid and accurate test for the diagnosis and prognosis of both VL and PKDL. Further, the assay provides an important molecular tool to monitor the efficacy of anti-leishmanial therapy or vaccines.
